# Regional effects of China’s monetary policy during the economic transition period: Based on China’s city classification system under the new normal

**DOI:** 10.1371/journal.pone.0291317

**Published:** 2023-09-13

**Authors:** Lingyu Tang, Guoju Ai, Zushun Cai

**Affiliations:** 1 School of Finance, Zhongnan University of Economics and Law, Wuhan, Hubei, China; 2 China Minsheng Bank Wuhan Branch, Wuhan, Hubei, China; 3 School of Finance, Zhejiang University of Finance and Economics, Hangzhou City, Zhejiang Province, China; East China Normal University, CHINA

## Abstract

We analyse the regional effects of China’s monetary policy during the economic transition period based on the city classification system under the New Normal. Using panel vector autoregressive (PVAR) models, we examine the differences in the influence of a unified monetary policy on four types of Chinese cities: first-tier, quasi-first-tier, second-tier, and third-tier cities, from perspective of economic output. Based on a comparative analysis of the results of impulse response function and variance decomposition, we conclude that whether for quantitative monetary policy or price-based monetary policy, the degree to which economic growth responds to the unified monetary policy varies across the four types of cities in the short and long terms. With reference to China’s economic transition under the New Normal, in the short term, this phenomenon probably relate to the varying degrees of financial marketization among the four city types; in the long run, this phenomenon may be attributed to the difference in the progress of industrial upgrading of these urban areas. Based on the analysis above, we suggest policy implications for Chinese cities based on the structural monetary policy, industrial upgrading, and market-oriented financial sector reforms.

## 1. Introduction

After decades of rapid economic growth, China has entered a phase of economic transition, referred to as the New Normal. As the largest emerging economy in the world, China continues to expand economically during the New Normal period, sustaining medium-high growth at a rate of around 6% per annum. Under the national policy of sustainable development, certain adjustments have been made to the strategies of economic development in this phase. Broadly, the overall development planning has progressively transitioned from a strategy of quantitative boost to the primary objective of improving quality [1]. The development philosophy has shifted from a growth-oriented model to structural adjustments [2]. In addition, China’s monetary policy reform was initiated during this economic stage. New structural and targeted principles have emerged for the formulation and implementation of the monetary policy, which is designed to achieve specific objectives of economic development under the New Normal [3]. One reason for this is that structural asymmetries often occur during the transmission process of a unified monetary policy. Note that a unified monetary policy cannot impact the economy in a simultaneous and equal way at the local level [4]. In fact, academia refers to the phenomenon of regional asymmetric transmission, caused by a unified monetary policy, as the regional effect of monetary policy.

Since 1949, China has experienced a strategic transformation from balanced regional economic development to unbalanced development and to coordinated development. Currently, China’s regional economy is moving towards the goal of coordinated development in the context of economic transition, which is in line with the inherent requirements of economic development to benefit the people ’s livelihood under the New Normal. As the central area of industrial development, cities have played a crucial role in China’s industrial upgrading process during the New Normal period. Obviously, the coordinated development of the urban economy has a significant impact on the success of China’s economic transition. Owing to comparative advantages and differences in resource endowments, the economies of various urban areas in China share a degree of complementarity. If the economic conditions and economic strength of various cities differ greatly from each other, the overall economic development of China will slow down, and this could be detrimental to the economic transition process. In addition, for a long time, China’s academia has focused on the regional impact of monetary policy. Evidently, the asymmetric transmission of a unified monetary policy among cities is also a non-negligible factor that hampers the coordinated development of the urban economy. Therefore, it is pragmatic to address the actual impact of a unified monetary policy on the economy of various cities. Based on this, we can take appropriate measures to deal with related economic issues to ensure the coordinated development of the urban economy and smooth economic transition. We attempt to further analyse the regional effects of China’s monetary policy on the urban economy during the economic transition and suggest appropriate policy implications on related issues, primarily to indicate some reference for the economic development of China and other emerging economies during the economic transition.

## 2. Literature review

Earlier, relevant research on the regional effects of monetary policy focused on the United States (U.S.), and this concept was first introduced by [5]. He observed that after the Federal Reserve conducted open market operations, a significant time-lag effect was evident in the transmission of the policy from New York to other states. The time-lag effect varied markedly, depending on the state. Subsequently, the Optimum Currency Area theory was proposed, and some scholars further enriched and refined it [6]. An optimum currency area is a geopolitical concept and refers to countries or regions that meet specific economic and financial conditions to establish a closely interlinked monetary policy framework, or even to use a single currency. This theory highlights that an optimum currency area is an ideal state of integration of the regional economy. In an optimum currency area, the regional asymmetric transmission of the unified monetary policy will diminish or even disappear. If an area is not an optimum currency area, this type of phenomenon will exist. We can infer from this theory that a tendency for a positive correlation exists between the degree of regional asymmetric transmission of a unified monetary policy and the difference in the conditions of economic development across various regions. Therefore, some scholars refer to this theory in zoning approaches to analyse the regional effects of monetary policy within a country or advocate the establishment of a particular currency area among regions with similar economic development. Later, during the 1990s, when various econometric models were derived, studies on the regional effects of monetary policy focused on quantitative analysis. Researchers had developed several econometric models to explain this phenomenon. At the empirical level, the regional effects of monetary policy became widespread. Using the vector autoregressive (VAR) model [7], verified that a unified monetary policy in the U.S. had different effects on regulating the economy across states. Another example is the application of the structural vector autoregressive (SVAR) model to show that a unified monetary policy has varying degrees of impact on economic fluctuations across U.S. regions [8].

Next, with the emergence of related studies in several countries, the research orientation can be broadly classified into two categories: (i) focus on a single country for conducting specific analysis on influencing factors, and (ii) explore such phenomena in different countries, mainly the European Union (EU) countries. Regarding the former, scholars have conducted research from the perspective of the credit channel, which is a critical channel with various influencing factors associated with the regional effects of monetary policy in a majority of countries. Studies have shown that influencing factors in relation to the credit channel within one country include the overall size of banks, the degree of reliance of local enterprises on the banking sector [9], regional concentration of banks, overall profitability and anti-risk ability in all banks, and non-bank credit financing efficiency [10]. In addition to the credit channel, different countries have their own individual channels. In Australia, some influencing factors are linked to the exchange rate channel [11]. For Canada, Indonesia, and South Korea, studies suggest that the regional asymmetric transmission of a unified monetary policy in these countries is associated with a host of factors in the interest rate channel [12–14]. Notwithstanding the influencing factors related to the various transmission channels of monetary policy, a few non-financial factors can also play a crucial role in the regional asymmetric transmission of a unified monetary policy in a country. For instance, the heterogeneity of industrial structures and demographic characteristics in different regions in Poland can contribute to this type of phenomenon [15].

The Optimum Currency Area theory came into practice with the establishment of the Euro zone. However, the practice has demonstrated that the employment of a unified currency and monetary policy in a non-optimum currency area does not guarantee the coordinated development of local economies. For EU member states, there are broader influencing factors related to such phenomena from a country-specific perspective. Apart from the aforementioned factors, some country-specific factors are also involved. In international trade, differences in the euro exchange rate, trade barriers, degree of openness, and international influence among member states play a major role in the varying dynamics of the unified euro policy on the economic growth of different states [16]. Moreover, at the economic level, differences in the distribution of industries, economic cycles, and diffusion of relevant economic information among EU member states are included [17–19].

In China, a related analysis on the regional effects of monetary policy was initially proposed by [20]. They concluded that under the mechanism of the market economy, a unified monetary policy often results in inconsistencies at the level of financial development across China’s regions, thus exacerbating the problem of imbalanced regional economic development. Since the 21st century, further studies have been conducted using quantitative analysis. Drawing on the thought of the Optimum Currency Area theory, Chinese scholars discussed this type of phenomenon on the basis of various zoning approaches, namely ‘three divisions’, ‘five divisions’, ‘eight divisions’ based on the eight economic zones, and ‘inter-provincial divisions’ based on administrative units [21–25]. Consistent with these findings, scholars advocate the formulation and implementation of differentiated monetary policies in various economic regions to further evolve China’s monetary policy framework and weaken imbalances in regional economic development. Subsequently, scholars attempted to analyse relevant influencing factors from the perspective of transmission channels. Broadly, both interest rate and credit channels are considered the two major channels with the most associated factors involved. These two channels are also the primary mechanisms of the regional asymmetric transmission of monetary policy in China [26].

According to existing studies, while some previous works have been devoted to this type of phenomenon, it needs further analysis. Scholars have studied the regional effects of China’s monetary policy. However, there are still some limitations in these studies. Compared to rural areas, China’s urban areas have a distinctly different financial environment, attributable to the urban-rural dual economic structure, which makes the transmission of China’s unified monetary policy between urban and rural areas inherently different [27]. However, existing research fails to separately discuss the regional effects of China’s monetary policy from the perspective of these two dimensions mentioned above, which has limited implications for further improving China’s monetary policy. In terms of the theoretical basis, the zoning approaches in current studies do not fully reflect the connotation of the optimum currency area. Take a research as an example, which is based on the ‘three divisions’ [22]. The eastern, central, and western regions separately represent the three economic zones of China from developed to underdeveloped. Undoubtedly, their economic development levels vary considerably. Exploring the regional effects of China’s monetary policy based on these ‘three divisions’ is generally consistent with the Optimum Currency Area theory. However, when analysing each region, we find that this is not the case. Even though the overall economic development of China’s western region is relatively backward, it includes some developed economic zones such as Chengdu, Chongqing, Xi’an, and Kunming. While the economic strength of the eastern region is stronger, there are some relatively underdeveloped economic districts in this region. Clearly, under the zoning approaches in existing studies, the economic development of districts within each region also varies considerably. If the analysis of the regional effects of China’s monetary policy is based only on the ‘three divisions’ or other zoning approaches mentioned above, the findings will not contribute to fully and effectively address the issues of economic development caused by the regional effects of China’s monetary policy, and fail to provide sufficient insights into ensuring the coordinated development of China’s regional economy. A similar case is observed in the European Union. To some extent, the establishment of the euro zone follows the Optimum Currency Area theory. However, the unified euro policy has not been effective in guaranteeing the coordinated development of European economies. A major reason for this is that, overall, even though the European Union economy is at a more developed level from a global perspective, there are significant gaps in member states’ level of economic strength. The unified euro policy has not been effective in ensuring the economic integration of Europe owning to not only the varying economic demands but also the different stages of economic development of member states. Specific analysis reveals that the Eurozone is still far from the theoretically optimum currency area. Member states such as Spain, France, and Luxembourg are among the world’s highly developed economies; however, Portugal and Greece are moderately developed, while Slovakia and Latvia have barely reached the standards of developed economies. In addition, when discussing the regional effects of China’s monetary policy, existing research does not separately analyse the magnitude of this policy based on the three distinct stages in China’s economic development: (i) adjustment period in the early days of the reform and opening up, (ii) the era of rapid growth, and (iii) the New Normal. China’s actual economic situation and development goals vary significantly during each stage of economic growth. However, existing research has failed to include these, therefore, the findings are less instructive for work on macroeconomic regulation. At last, existing research has not sought the inherent reasons that underpin this type of phenomenon but has only looked for relevant influencing factors from the perspective of the monetary policy transmission channel, which is limited to the surface of the phenomenon. Thus, when giving corresponding policy recommendations based on their findings, existing research remains at the level of perfecting China’s monetary policy framework, rather than considering the inherent reasons underlying it. In summary, to study the regional effects of China’s monetary policy in a more rational way, it is advisable to improve on the existing studies to effectively address the related issues caused by the regional asymmetric transmission of China’s unified monetary policy.

In view of the above, we conduct further research on the regional effects of China’s monetary policy based on the following improvements. First, the time range of our discussion is confined to the New Normal. We analyse the regional effects of China’s monetary policy during this transition period, attempting to make our findings more instructive to the work on macroeconomic regulation. In addition, we separate urban and rural areas to discuss this phenomenon only from the perspective of the urban economy, avoiding the limited implications of our findings due to the distinctly different financial environment of urban and rural areas. Then, We explore the regional asymmetric transmission of a unified monetary policy among Chinese cities based on the city classification system under the New Normal. In this classification system, within the same city type, the economic conditions and economic strength are almost at the same level. Thus, the varying degrees of their economic development are almost similar, which approximates the Optimum Currency Area theory in comparison to other zoning approaches. According to this system, we can study and address issues resulting from the regional effects of China’s monetary policy appropriately, avoiding problems such as those in the Eurozone. Finally, the inherent reasons underlying this phenomenon must be assessed. We will cope with related issues and suggest appropriate policy implications, rather than merely limiting ourselves to addressing them by perfecting China’s monetary policy framework, as has been done in existing studies. And due to space limitations, we have not discussed the regional effects of monetary policy from other aspects (e.g. employment, industry differences, etc.), but only from perspective of economic output. We will further address these in the future studies.

## 3. Research design

### 3.1. Description of city division and sample lists of four types of cities

The two official classification systems for Chinese cities are administrative and population size. However, in terms of the economic conditions and economic strength, these two classifications cannot accurately reflect the economic development of cities. To better measure the comprehensive level of economic development, the general public and some mainstream media houses in China, referring to the international classification of global cities, propose a new classification system based on the economic strength of cities since the New Normal, and this is widely recognised in China. Under this new classification system, Chinese cities are classified into six types: first-tier cities, quasi-first-tier cities, second-tier cities, third-tier cities, fourth-tier cities, and fifth-tier cities, in descending order of economic strength. This city classification system has been broadly referred to and employed in studies on China’s urban economy, such as [28, 29]. Among the various lists released under this classification approach, the city division lists issued by the China Business Network (CBN) media are the most authoritative. Compared to administrative and population size classification, this city classification system is a real effort to group cities with nearly equal economic strength. Therefore, we selected this classification system to explore the regional effects of China’s monetary policy at the city-level. Considering the availability and quality of data, we chose four groups of first-tier cities, quasi-first-tier cities, second-tier cities, and third-tier cities for research. For Chinese cities that are economically more backward (e.g. fourth-tier and fifth-tier cities cities), the GDP data of many cities may be adulterated and overestimated to a certain degree. Therefore we do not add them as subjects to this study. Given that the cut-off year of data for every indicator involved in this study was 2019, we selected all 119 samples of cities as research objects according to the lists published by CBN Media in 2019, as presented in Table 1.

**Table 1:** Classification list of first-tier, quasi-first-tier, second-tier, and third-tier cities.

City Type	Number	Name
First-tier cities	4	Beijing, Shanghai, Guangzhou, Shenzhen
Quasi-first-tier cities	15	Chengdu, Hangzhou, Chongqing, Wuhan, Suzhou, Tianjin, Nanjing, Xi’an, Qingdao, Shenyang, Ningbo, Changsha, Zhengzhou, Dongguan, Kunming
Second-tier cities	30	Wuxi, Foshan, Zhuhai, Hefei, Dalian, Fuzhou, Xiamen, Jinan, Wenzhou, Harbin, Nanning, Changchun, Quanzhou, Shijiazhuang, Guiyang, Nanchang, Jinhua, Changzhou, Nantong, Jiaxing, Taiyuan, Xuzhou, Huizhou, Zhongshan, Taizhou, Yantai, Lanzhou, Shaoxing, Haikou, Yangzhou
Third-tier cities	70	Shantou, Huzhou, Yancheng, Weifang, Baoding, Zhenjiang, Luoyang, Taizhou, Urumqi, Linyi, Tangshan, Zhangzhou, Ganzhou, Langfang, Hohhot, Wuhu, Guilin, Yinchuan, Jieyang, Sanya, Zunyi, Jiangmen, Jining, Putian, Zhanjiang, Mianyang, Huai’an, Lianyungang, Zibo, Yichang, Handan, Shangrao, Liuzhou, Zhoushan, Xianyang, Jiujiang, Hengyang, Weihai, Ningde, Fuyang, Zhuzhou, Lishui, Nanyang, Xiangyang, Daqing, Cangzhou, Xinyang, Yueyang, Shangqiu, Bengbu, Zhaoqing, Qingyuan, Chuzhou, Longyan, Jingzhou, Xinxiang, Anshan, Xiangtan, Maanshan, Sanming, Chaozhou, Meizhou, Qinhuangdao, Nanping, Jilin, Anqing, Tai’an, Suqian, Baotou, Chenzhou

Source: China Business Network (CBN)

### 3.2. Selection of variables

In view of data availability and rationality of the variables, we select the GDP growth rate as a proxy variable to measure the level of economic growth of each Chinese city, denoted as *RGDP*. Specific values are measured according to the officially released GDP data in two successive years of a city. As China’s economy entered the period of New Normal around 2012, and the city classification system has been widely accepted in China from this year, the starting year of data for all variables was 2012. The data type for this variable was annual data from 2012 to 2019. Table 2 presents detailed information on this variable. Specifically, as a result of the Fourth National Economic Census of China, the GDP estimate for some cities changed marginally in the statistical data since 2018. After releasing the relevant data in 2018 under the previous statistical calibre, data for these cities in this year were revised consistent with the new statistical calibre, and the corresponding revision notice was issued by the Bureau of Statistics of each city. Owing to the two different statistical standards, the GDP data of these cities may have had two different values in 2018. Therefore, when calculating the value of *RGDP*, we first verify the consistency of the statistical calibre of the GDP data of each city in two successive years. The value of *RGDP* for each city was then measured based on the same standard of the statistical calibre to ensure the rationality of the sample data.

**Table 2:** Detailed information for each variable.

Variable Name	Symbol	Economic Implication	Original Data Source
GDP growth rate	*RGDP*	Economic growth level of each city	China National Bureau of Statistics Database, the Bureau of Statistics of each city, and China Economic Information Network (CEINET) Statistics Database
Natural logarithm of money supply (M2)	*logM2*	Actual implementation of quantitative monetary policy	China National Bureau of Statistics Database
Weighted interbank offered rate	*WIBOR*	Actual implementation of price-based monetary policy	Economy Prediction System (EPS) Database

Considering that the definite mechanisms of regional asymmetric transmission of monetary policy in China are mainly through the credit and interest rate channels [26], and the implementation of China’s monetary policy is a combination of quantitative and price-based monetary policy, we follow the common practices applied by scholars when studying the regional effects of China’s monetary policy, separately selecting one indicator from the quantitative and price-based monetary policy indicators as the respective proxy variables. The selected indicators were the intermediate targets. For quantitative monetary policy indicators, broad money supply (M2) is selected as a proxy variable, which is proven to be one of the most critical indicators of the credit channel of China’s monetary policy. To eliminate heteroskedasticity and the problem that the order of magnitude of this variable is too far from others in the model, we performed logarithmic processing on this variable, and the processed variable is denoted as *logM*2. The annual data span for this variable was from 2012 to 2019. Detailed information is shown in Table 2. For price-based monetary policy indicators, as China’s market economic system has gradually improved in the New Normal, an indicator related to the interest rate channel with a high degree of marketisation is selected as a proxy variable. Specifically, we selected the annual 60-day Weighted Interbank Offered Rate indicator among Chinese commercial banks as a proxy variable, denoted as *WIBOR*. The annual span of data for the variable is from 2012 to 2019, and its values are weighted according to the monthly value and corresponding trading volume.

### 3.3. Research hypotheses

Consistent with the current monetary policy theory, owing to the price stickiness effect, the monetary authority can regulate the level of economic growth for a duration through the formulation and implementation of monetary policy by stimulating the change in aggregate demand. And the definite mechanisms transmission of monetary policy in China are mainly through the credit and interest rate channels. Commonly, its path of influence is dynamic. In light of the above, we propose hypothesis 1.

Hypothesis 1: Under the New Normal, there is a dynamic relationship between the two aggregate monetary policy variables and the variable *RGDP* in the four types of cities.

Typically, money supply is not entirely exogenous. According to the prevailing monetary policy theories, as well as a large number of existing relevant empirical studies, it has been shown that there is often a two-way causal relationship between monetary policy and the level of economic growth in a country. So there are often interactive connections between monetary policy indicators and various primary indicators of economic growth. Thus, Hypothesis 2 is proposed.

Hypothesis 2: There is a two-way causal link between the two aggregate monetary policy variables and the variable *RGDP* in the four city models.

A number of existing studies have shown that the phenomenon of regional asymmetric transmission of a unified monetary policy is prevalent. In addition, a remarkable regional heterogeneity in economic development exists among the four types of cities. From the view of economic theory, regional heterogeneity is one of the fundamental cause of the regional effects of monetary policy. And the four types of cities are not a theoretically optimum currency area, which is likely to make the asymmetric transmission of a unified monetary policy among these cities.

Therefore, hypothesis 3 is proposed.

Hypothesis 3: The impact of the two aggregate monetary policy variables on the variable *RGDP* among the four city models is not the same.

### 3.4. Model selection and construction

In view of the two-way causal relationship and dynamic impact path between monetary policy indicators and economic growth, several existing studies applied VAR-type models for empirical analysis on such economic issues. In this study, the data types for the three selected variables are panel data. Therefore, we selected the PVAR model for the empirical analysis [30]. According to the selected variables, the preliminary simultaneous formulas are established as follows:

$$RGDP_{it}=\sum_{j=1}^{p}\left(\alpha_{11}^{\left(j\right)}RGDP_{i,t-j}+\alpha_{12}^{\left(j\right)}logM2_{t-j}+\alpha_{13}^{\left(j\right)}WIBOR_{t-j}\right)\;+\delta_{1t}+\varepsilon_{1i,t}$$ (1)

$$logM2_{t}=\sum_{j=1}^{p}\left(\alpha_{21}^{\left(j\right)}RGDP_{i,\;t-j}+\alpha_{22}^{\left(j\right)}logM2_{t-j}+\alpha_{23}^{\left(j\right)}WIBOR_{t-j}\right)+\delta_{2t}+\varepsilon_{2i,t}$$ (2)

$$\;WIBOR{\;}_{t}=\sum_{j=1}^{p}\left(\alpha_{31}^{\left(j\right)}RGDP_{i,\;t-j}+\alpha_{32}^{\left(j\right)}logM2_{t-j}+\alpha_{33}^{\left(j\right)}\;WIBOR{\;}_{it-j}\right)+\delta_{3t}+\varepsilon_{3i,t}$$ (3)

In the above formulas, *RGDP*_*it*_, *logM*2_*it*_, *WIBOR*_*it*_ separately represent the values of the cross-sectional individual i of each variable at time t; $\alpha_{11}^{\left(j\right)}$, $\alpha_{12}^{\left(j\right)}$, $\alpha_{13}^{\left(j\right)},\alpha_{21}^{\left(j\right)},\alpha_{22}^{\left(j\right)},\alpha_{23}^{\left(j\right)},\alpha_{31}^{\left(j\right)},\alpha_{32}^{\left(j\right)},\alpha_{33}^{\left(j\right)}$ separately represent the coefficients to be estimated; *RGDP*_*i*, *t-j*_, *logM*2_*t-j*_, *WIBOR*_*t-j*_ separately indicate the j-th order lag term of each variable; *δ*_1*t*_, *δ*_2*t*_, *δ*_3*t*_ separately represent the time-point fixed effects; *ε*_1*i*_, _*t*_, *ε*_2*i*_, _*t*_, *ε*_3*i*_, _*t*_ separately represent the random disturbance terms. According to the above simultaneous formulas, we rewrite it in the matrix form of the PVAR(p) model:

$$Y_{it}=\sum_{j=1}^{p}\varphi_{j}Y_{i,t-j}+\delta_{t}+\epsilon_{i,t}$$ (4)

In (4), *$Y_{it}=\left(\begin{array}{c} RGDP_{it}\\ logM2_{t}\\ \;WIBOR{\;}_{t} \end{array}\right)$, $Y_{i,\;t-j}=\left(\begin{array}{l} RGDP_{i,t-j}\\ logM2_{it-j}\\ WIBOR_{t-j} \end{array}\right)$, $\varphi_{j}=\left(\begin{array}{c} \begin{array}{ccc} \alpha_{11}^{\left(j\right)} & \alpha_{12}^{\left(j\right)} & \alpha_{13}^{\left(j\right)} \end{array}\\ \begin{array}{ccc} \alpha_{21}^{\left(j\right)} & \alpha_{22}^{\left(j\right)} & \alpha_{23}^{\left(j\right)} \end{array}\\ \begin{array}{ccc} \alpha_{31}^{\left(j\right)} & \alpha_{32}^{\left(j\right)} & \alpha_{33}^{\left(j\right)} \end{array} \end{array}\right)$ , $\delta_{t}=\left(\begin{array}{c} \delta_{1t}\\ \delta_{2t}\\ \delta_{3t} \end{array}\right)$, $\epsilon_{i,\;t}=\;\left(\begin{array}{c} \begin{array}{cc} \varepsilon_{1i,} & {}_{t} \end{array}\\ \begin{array}{cc} \varepsilon_{2i,} & {}_{t} \end{array}\\ \begin{array}{cc} \varepsilon_{3i,} & {}_{t} \end{array} \end{array}\right)$*

According to Eq (4), and based on the four respective types of cities, we establish four respective PVAR models in the next chapter.

## 4. Empirical analysis

### 4.1. Descriptive analysis of variables

The following descriptive analysis is showed for the statistical characteristics of each variable, as shown in Table 3. We can see that the order of magnitude differences between the variables are all within 10^3^, which indicates that the regression results are not biased by the order of magnitude differences. In addition, it should be noted that a few sample observations of the variable *RGDP* are outliers owing to the individual statistical data shrinkage. Outliers were excluded from the subsequent regression analysis.

**Table 3:** Descriptive analysis.

Variable	Mean	Standard Error	Minimum	Maximum	Sample Size
*RGDP* (first-tier city)	0.094	0.024	0.037	0.154	32
*RGDP* (quasi-first-tier city)	0.095	0.049	-0.237	0.244	120
*RGDP* (second-tier city)	0.090	0.041	-0.119	0.229	240
*RGDP* (third-tier city)	0.081	0.058	-0.405	0.209	560
*logM*2	14.127	0.213	13.789	14.418	Depending on the model
*WIBOR*	0.042	0.006	0.032	0.051	Depending on the model

### 4.2. Selection of number of lag orders

Short panel data are used in this study. In each model, the number of periods t = 8 < 10. Especially, second- and third-tier city models, the number of samples in each section N ≫ T. If conventional information criteria such as MMSCaic, MMSCbic, and MMSChqic are applied for selecting the number of lag orders, the fitting results of the information statistics may be biased or cannot be fitted. Therefore, consistent with current scholars’ practice of dynamic modelling, which is characterised by short panel data, the number of lag orders of the four models is uniformly set to one order. The following PVAR model settings were carried out:

$$\;1.\;\text{First}\;-\;\text{tier}\;\text{city}:\;{\text{Y}}_{\text{it}}{=\varphi }_{\text{1}}{\text{Y}}_{\text{i,}\;\text{t-1}}{+\delta }_{\text{t}}\text{+}\in_{\text{i,}\;\text{t}}$$ (5)

i = 1,…,4; t = 1,…,8

$$\;2.\;\text{Quasi}\;-\;\text{first}\;-\;\text{tier}\;\text{city:}\;{\text{Y}}_{\text{it}}{=\varphi }_{\text{1}}{\text{Y}}_{\text{i,}\;\text{t-1}}{+\delta }_{\text{t}}\text{+}\in_{\text{i,}\;\text{t}}$$ (6)

i = 1,…,15; t = 1,…,8

$$\;3.\;\text{Second}\;-\;\text{tier}\;\text{city:}\;{\text{Y}}_{\text{it}}{=\varphi }_{\text{1}}{\text{Y}}_{\text{i,}\;\text{t-1}}{+\delta }_{\text{t}}\text{+}\in_{\text{i,t}}$$ (7)

i = 1,…,30; t = 1,…,8

$$\;4.\;\text{Third}\;-\;\text{tier}\;\text{city:}\;{\text{Y}}_{\text{it}}{=\varphi }_{\text{1}}{\text{Y}}_{\text{i,}\;\text{t-1}}{+\delta }_{\text{t}}\text{+}\in_{\text{i,t}}$$ (8)

i = 1,…,70; t = 1,…,8

### 4.3. Parameter estimation

After selecting the number of lag orders, the parameter estimation is to be performed. First, considering that some observations in the GDP of a few cities experienced a certain degree of statistical bubbles during certain years, several sample observations with distinct statistical bias have been removed (e.g. Shenyang in 2016, Dalian in 2016, and Baotou in 2017). Specifically, we eliminate the observations of RGDP in a year where the range of change in its value is more than 15% compared to the previous year, and the cross-sectional sample cities with data quality is severely distorted due to several statistical bubbles during a few years. For the latter, the section samples of Shenyang in the quasi-first-tier city model and Baotou in the third-tier city model are excluded. Having completed the preparatory works above, the parameters in each model are estimated using the generalised system moment (SYSTEM-GMM) based on the processed unbalanced panel data. Table 4 presents the regression results.

**Table 4:** Parameter estimation results of the four models.

Model	Explanatory Variable		Explained Variable		
			*WIBOR*	*RGDP*	*logM*2
First-tier city		*WIBOR L*1.	-0.448*** (-3.21)	-2.573*** (-4.01)	-0.087 (-0.27)
		*RGDP L*1.	0.150*** (3.54)	-0.040 (-0.19)	-0.293*** (-3.26)
		*logM*2 *L*1.	-0.028*** (-5.70)	-0.084*** (-3.72)	0.921*** (121.09)
Quasi-first-tier city		*WIBOR L*1.	-0.371*** (-4.08)	-2.586*** (-5.29)	-0.282 (-1.42)
		*RGDP L*1.	0.080*** (3.71)	0.202** (2.55)	-0.125*** (-3.63)
		*logM*2 *L*1.	-0.027*** (-8.47)	-0.032** (-2.05)	0.919*** (183.40)
Second-tier city		*WIBOR L*1.	-0.333*** (-5.02)	-2.598*** (-4.69)	-0.376*** (-2.73)
		*RGDP L*1.	0.0593*** (3.99)	0.433*** (3.86)	-0.072*** (-2.17)
		*logM*2 *L*1.	-0.027*** (-11.75)	-0.039*** (-2.78)	0.920*** (261.14)
Third-tier city		*WIBOR L*1.	-0.268*** (-6.33)	-0.610* (-1.81)	-0.463*** (-5.39)
		*RGDP L*1.	0.037*** (2.50)	0.299*** (2.86)	-0.047* (-1.92)
		*logM*2 *L*1.	-0.027*** (-13.69)	-0.026** (-2.09)	0.919*** (321.36)

Note: standard errors are in parentheses.

*** p < 0.01

** p < 0.05

* p < 0.1.

### 4.4. Model robustness test

**Fig 1:**
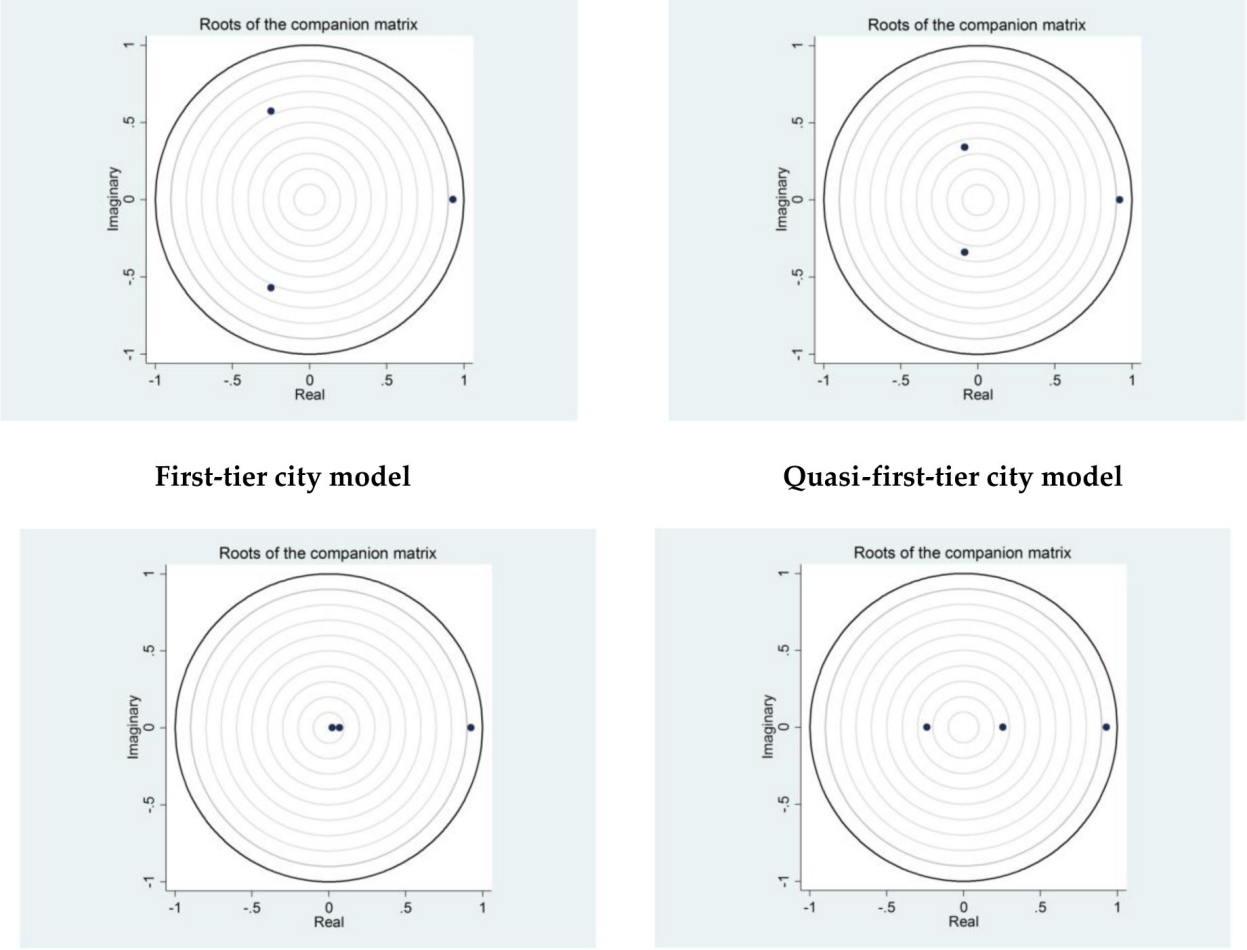
Complex plane images of the unit circle of eigenvalues in each model.

Fig 1 shows the results of the model stability test. It can be intuitively seen that, for the four PVAR models, each root falls within the unit circle in the complex plane, which indicates that all the models are robust. At the same time, hypothesis 1 is tested among the four models.

### 4.5. Granger causality test

Table 5 presents the results of the Granger causality test. In the report, except in the models of first-tier and quasi-first-tier cities, the interpretation strength of *logM*2 by *WIBOR* fails to pass the test of confidence level of less than 10%, and the Chi2 statistics of all excluded variables on the formula variables reject the original hypothesis at the confidence level of less than 10% in each model. This means that in each of the four PVAR models, two monetary policy variables and the urban economic growth variable maintain a two-way Granger causality relationship with each other. Hypothesis 2 is verified among the four models.

**Table 5:** Results of Chi2 statistics in the Granger causality test.

Ho: Excluded variables are not the Granger reason for the formula variables				
Model	Excluded variable	Formula variables		
		*WIBOR*	*RGDP*	*logM*2
First-tier city	*WIBOR*	-	16.057***	0.071
	*RGDP*	12.518***	-	10.598***
	*logM*2	32.511***	13.848***	-
	*WIBOR*,*RGDP*	-	-	14.289***
	*WIBOR*,*logM*2	-	21.073***	-
	*RGDP*,*logM*2	67.714***	-	-
Quasi-first-tier city	*WIBOR*	-	27.976***	2.025
	*RGDP*	13.734***	-	13.178***
	*logM*2	71.707***	4.188**	-
	*WIBOR*,*RGDP*	-	-	17.969***
	*WIBOR*,*logM*2	-	28.571***	-
	*RGDP*,*logM*2	144.381***	-	-
Second-tier city	*WIBOR*	-	22.037***	7.427***
	*RGDP*	15.886***	-	4.689**
	*logM*2	138.100***	7.732***	-
	*WIBOR*,*RGDP*	-	-	16.950***
	*WIBOR*,*logM*2	-	22.047***	-
	*RGDP*,*logM*2	226.066***	-	-
Third-tier city	*WIBOR*	-	3.287*	29.104***
	*RGDP*	6.226**	-	3.690*
	*logM*2	187.503***	4.356**	-
	*WIBOR*,*RGDP*	-	-	31.549***
	*WIBOR*,*logM*2	-	5.093*	-
	*RGDP*,*logM*2	582.447***	-	-

Notes: ***, **, and * indicate that the Chi2 statistics reject the null hypothesis at the confidence levels of 1%, 5%, and 10%, respectively.

### 4.6. Impulse response function analysis

As the perturbation terms between formulas in a model may be correlated, orthogonal impulse response functions are selected. First, the variables are sorted in the order of their relative exogeneity: *RGDP*, *logM*2, *WIBOR*. Consistent with this order, orthogonal impulse response functions are separately performed in the four models. After the initial analysis, the order of the variables are altered several times, and orthogonal impulse response functions are correspondingly reused for the robustness test. The conclusions suggest that with the order of the three variables being adjusted, the trends of the paths of impact between variables in various results remain consistent. Thus, the simulation results are comparatively robust. In view of these, as well as space limitations, we do not show the results of other orders but only list the results executed in the order set for the first time. To ensure the stability of the results, 500 simulations are carried out during the simulation process using Monte Carlo with a confidence interval of 95% and a seed value of 20,000. A time span of 0–10 phases is set for the simulations.

**Fig 2:**
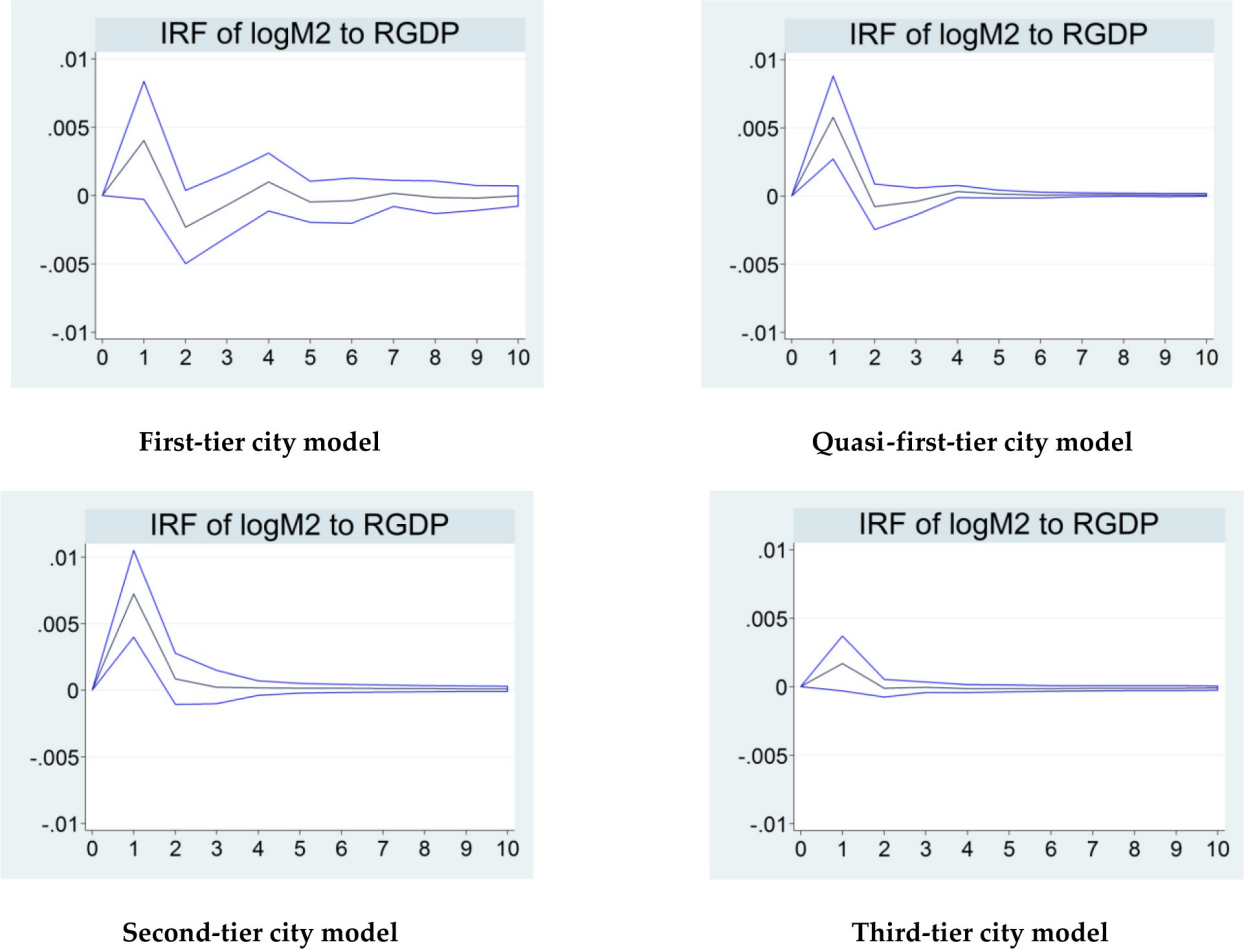
Impact of the variable *logM*2 on *RGDP* at different points in time in each model.

**Fig 3:**
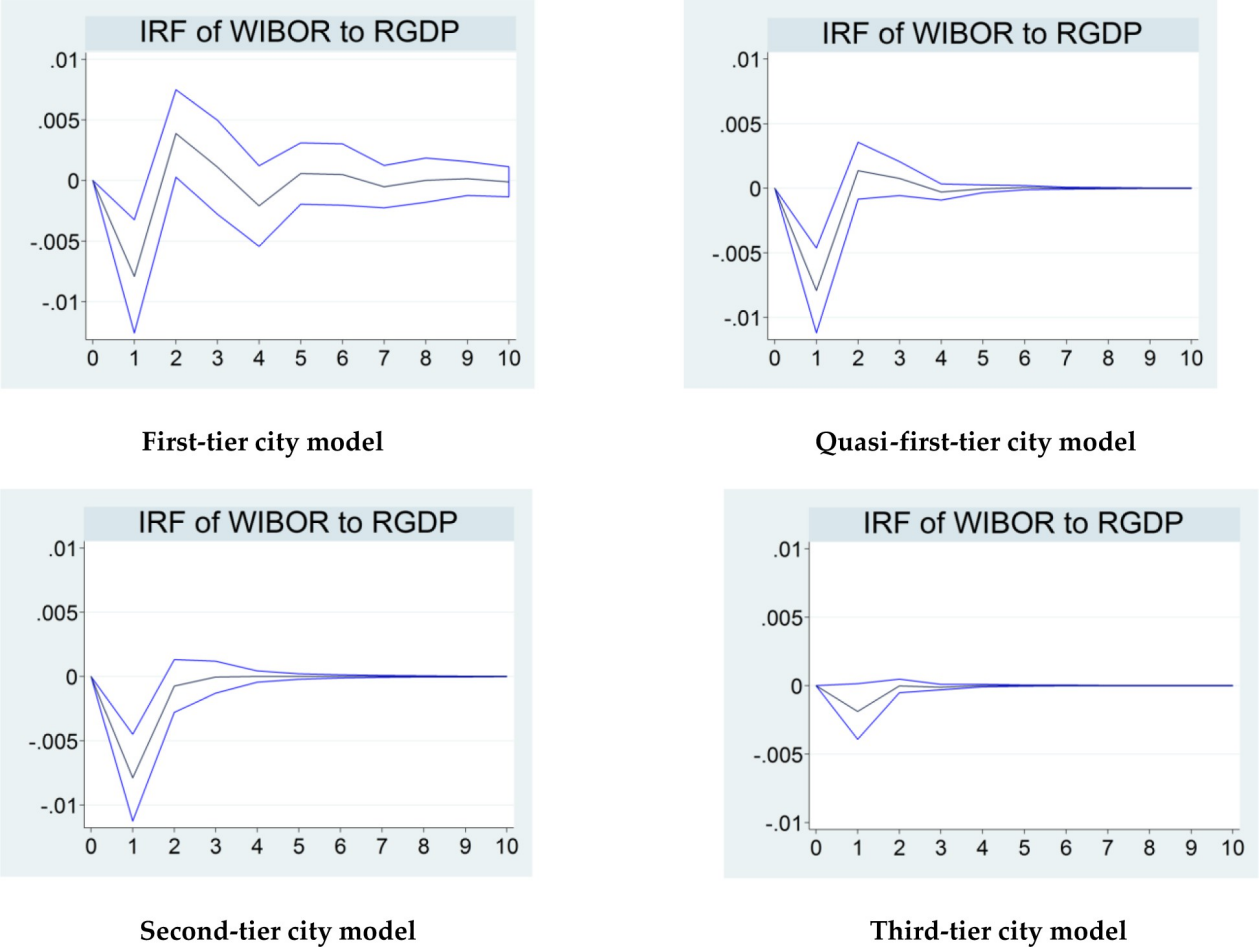
Impact of the variable *WIBOR* on *RGDP* at different points in time in each model.

Figs 2 and 3 individually demonstrate the paths of change of the variable *RGDP* under the respective impact of the *logM*2 and *WIBOR* in the four models. In Fig 2, when impacted by *logM*2, the value of *RGDP* has an immediate positive change in each model, and the paths of change are broadly parallel in all four models. More specifically, the value exhibits a definite linear increase and peaks at the end of the first phase, then decreases to a trough at the end of the second phase, generally around zero, and finally oscillates gradually back to zero. These results suggest that in the four models, when the variable *logM*2 receives an exogenous shock, it gives an immediate positive impact to *RGDP* over time, which is consistent with the price stickiness effect in the short term in the current monetary policy theory. In addition, the variable *logM*2 does not have the same degree of impact on *RGDP* in the four models. Overall, in the first-tier, quasi-first-tier, and second-tier city models, the degrees of impact are generally equivalent and distinctly higher than that in the third-tier city model. In particular, the values of the unit of change of the variable *RGDP* in the first three city models are all around 0.005 at the peak, while in the third-tier city model, it is considerably lower than 0.005.

In Fig 3, when impacted by *WIBOR*, the *RGDP* value presents a changing trend in each model, and the overall paths of change are also nearly parallel. Specifically, the value of *RGDP* first plummets and bottoms out at the end of the first phase, then rises to nearly zero at the end of the second phase, and ultimately oscillates gradually back to zero. Similar to Fig 2, the results of the simulations in Fig 3 are also consistent with the price stickiness effect in the short term in the current monetary policy theory, which suggests that price stickiness tends to lead to a negative short-term linkage between the indicators of price-based monetary policy and the indicators of the level of economic growth. In addition, there are some differences in the degrees of impact of the four different models. The degrees of impact of *WIBOR* on *RGDP* in the first-, quasi-first-, and second-tier city models are roughly equivalent, all significantly higher than those in the third-tier city model. In particular, the values of the unit of change of the variable *RGDP* in the first three city models are distinctly below -0.005 at the end of the first phase. In contrast, in the third-tier city model, this value is much higher than -0.005.

In summary, the simulation results indicate that both monetary policy variables *WIBOR* and *logM*2 separately have a certain degree of impact on *RGDP* in all four models in the short term during the New Normal period. While the paths of the impact in the four models are broadly similar, the degrees are not precisely the same. More specifically, the extents of the short-term impact of the two aggregate monetary policy variables on *RGDP* in the first-tier, quasi-first-tier, and second-tier city models are broadly at the same level and significantly higher than that in the third-tier city model. Thus, Hypothesis 3 is verified. Based on the above results, we infer that these results are related to the varying degrees of development of cities’ financial industries. In China, there is often a positive correlation between the development of the financial industry and the degree of impact of monetary policy on the economy across regions in the transmission process in the short term [31]. Broadly, first-tier, quasi-first-tier, and second-tier cities belong to the categories of capital city, direct-controlled municipalities, provincial capital cities, municipalities with Independent Planning Status, and regional economically strong cities in China. In fact, under the New Normal, their financial industry is well-developed, and values for the majority of financial development indicators are significantly higher than those of other cities in China. In contrast, the financial industry in third-tier cities is relatively underdeveloped during this period of economic transition. So the results of impulse response function above are likely to be caused by the varying degrees of development of the financial industry in these cities. Further analysis shows that under the influence of the trend of economic transition, China’s financial industry reached the market-oriented reform stage during the New Normal period. Consequently, the degree of development of the financial industry among cities depends largely on the progress of financial marketisation under the New Normal. Compared to third-tier cities, the first three types of cities had relatively higher degrees of financial marketisation during this period. And the progress of financial marketisation of third-tier cities remains relatively slow due to local protection and market entry barriers. Synthesising the above analysis, we presume that the varying degrees of financial marketisation among the four types of cities is the main explanation for the impulse response function results.

### 4.7. Variance decomposition

Table 6 shows the results of variance decomposition in each model. For the four models, regardless of whether the number of phases is 10 or 20, both monetary policy variables have definite cumulative explanatory strength for *RGDP*, which indicates that, China’s unified monetary policy will have a cumulative contribution to the economic growth of the four types of cities in the long run. To further analyse, with the city models varying, there are notable differences in values of the cumulative explanatory strength of the two monetary policy variables to *RGDP*, which further validates the asymmetric transmission of a unified monetary policy across the four types of cities in the long run. In terms of the results of variance decomposition, the cumulative explanatory strengths of *WIBOR* and *logM*2 jointly on *RGDP* in the 20th phase are 22.55%, 22.77%, 10.96%, and 0.96% in the first-tier, quasi-first-tier, second-tier, and third-tier city models, respectively. Evidently, in the long run, the two monetary policy variables make the highest contribution to *RGDP* in the first-tier city and the quasi-first-tier city models, while they make an intermediate contribution to *RGDP* in the second-tier city model and a lower contribution to the third-tier city model. Thus, Hypothesis 3 is further verified. Reflecting on the variance decomposition results, we infer that these results are associated with the varying degrees of development of cities’ productivity. According to [32], the difference in the degree of development of regional productivity is a key factor in the long-term impact of monetary policy on the economy. Evidently, the degrees of productivity development in the first-tier, quasi-first-tier, second-tier, and third-tier cities are in decreasing order, which, according to the above empirical results, shows a roughly positive correlation with the degrees of impact of a unified monetary policy on the economic growth level of the four types of cities in the long run. Therefore, the above results are likely to be the outcome of the varying degrees of development in cities’ productivity. Further analysis shows that the development of China’s economy was in a process of transition during the New Normal period. Industrial upgrading has became a primary economic objective during this period of economic transition. Meanwhile, the degree of productivity development among cities is closely related to the progress of industrial upgrading. In terms of the size and number of high-tech enterprises, the level of R&D, and the overall share of tertiary industry, the strength of first-tier cities and quasi-first-tier cities in the above aspects are obviously stronger than those of second- and third-tier cities. In view of the above, we consider that, in the long run, the difference in the progress of industrial upgrading of the four types of cities may be the main factor for the asymmetric impact of the unified monetary policy on the level of economic growth of the four types of cities under the New Normal.

**Table 6:** Variance decomposition report.

Model	Impulse Variable		Phase		Response Variable
		*S*		*RGDP*	
First-tier city	*logM*2	10		4.89%	
	*WIBOR*	10		17.66%	
	*logM*2	20		4.89%	
	*WIBOR*	20		17.66%	
Quasi-first-tier city	*logM*2	10		7.83%	
	*WIBOR*	10		14.94%	
	*logM*2	20		7.83%	
	*WIBOR*	20		14.94%	
Second-tier city	*logM*2	10		5.03%	
	*WIBOR*	10		5.92%	
	*logM*2	20		5.04%	
	*WIBOR*	20		5.92%	
Third-tier city	*logM*2	10		0.43%	
	*WIBOR*	10		0.52%	
	*logM*2	20		0.44%	
	*WIBOR*	20		0.52%	

## 5. Conclusion and policy implications

### 5.1. Conclusion

Based on this discussion, we make the following conclusions. For both quantitative monetary policy and price-based monetary policy, economic growth response to the unified monetary policy varies across the four types of cities in both the short and long term during the New Normal period. In terms of the empirical analysis results, in the long run, the variables *WIBOR*, a proxy variable for price-based monetary policy indicators, and *logM*2, a proxy variable for quantitative monetary policy indicators, have the strongest impact on the variable *RGDP*, a proxy variable for the level of economic growth, in the first- and quasi-first-tier cities, while there is a moderate impact on the variable *RGDP* in the second-tier cities and a weaker impact in the third-tier cities. This may be attributed to the difference in the progress of industrial upgrading among the four types of cities. Moreover, in the short term, the impact of the two variables of monetary policy on the variable *RGDP* in the first-tier, quasi-first-tier, and second-tier cities are equivalent, but distinctly stronger than those in the third-tier cities, which is likely to be related to the varying degrees of financial marketisation among the four types of cities.

### 5.2. Policy implications

Based on the conclusions, and considering the background of economic transition and the principles of the formulation and implementation of China’s monetary policy under the New Normal, the policy implications are given below, which is also to provide some corresponding references for emerging economies with medium-high economic growth in Asia and Latin America.

1. The strategy of promoting industrial upgrading in economic development should be expanded in relatively underdeveloped urban areas, such as second- and third-tier cities. China must adopt appropriate economic policies to stimulate the establishment and expansion of high-tech enterprises and R&D investment in these cities. Specific policies may include financial and fiscal support, tax incentives, talent introduction, establishment of targeted public R&D platforms, and the informatization of traditional industries. From a long-term perspective, we can fundamentally deal with the issues of the asymmetric transmission of a unified monetary policy on the output levels among cities under the New Normal by accelerating the progress of industrial upgrading in underdeveloped cities.
2. The reform progress of financial marketisation needs to be deepened in urban areas that have relatively underdeveloped economic conditions, such as third-tier cities. In these cities, the lag in market-oriented reforms of the financial industry have led to financial barriers. By further promoting the reform progress of financial marketisation under the New Normal, we can make the transmission channel of monetary policy unobstructed among these cities, and thereby better facilitate financial support for the urban economy. Specific policies may include reducing barriers to market access in the financial industry, liberalising interest rate control, improving the autonomy of banking operations, and increasing the proportion of private banks in the banking industry.
3. Depending on the varying levels of economic development of Chinese cities, monetary authorities can attempt to implement hierarchical regulations at the city level. Differentiated monetary policies should be implemented among cities through various structural monetary policy tools, such as Mortgage Supplementary Loans (PSL), standing lending facilities (SLF), medium-term lending facilitation (MLF), and central bank bill swaps (CBS). For cities with a reasonably well developed economy, such as first- and quasi-first-tier cities, prudent monetary policies can be locally implemented to keep the economic development of these urban areas steady and progressive, preventing overheating of the economy and the appearance of excessive economic bubbles in these cities. For cities with a relatively underdeveloped economy, such as third-tier cities, loose monetary policies can be locally implemented to reinforce their overall economic development.

## Supporting information

S1 Data (ZIP)
